# Preclinical evaluation of lateral interbody fusions using 3D printed PEEK or 3D printed titanium cages

**DOI:** 10.1016/j.xnsj.2025.100756

**Published:** 2025-07-03

**Authors:** William Robert Walsh, Matthew Pelletier, Dan Wills, Tian Wang, Max Lloyd, Michael Veldman, Nick Cordaro, Mark Brady

**Affiliations:** a Surgical and Orthopaedic Research Laboratories, UNSW Sydney, Sydney, Australia; b Invibio Ltd. Hillhouse International, Thornton-Cleveleys, FY5 4QD, United Kingdom; c SpiTrex 3D, Carlsbad, CA, United States

**Keywords:** Interbody fusion, Additive manufacturing, 3D printed PEEK, 3D printed Titanium, Preclinical, Bone, Histology, Large animal model

## Abstract

**Background:**

PEEK interbody cages are well established. 3D porous PEEK designs can now be produced with additive manufacturing. This study compared the in-vivo response of additive manufactured porous PEEK (3D PEEK) and titanium alloy (3D Ti) cages.

**Methods:**

Interbody fusion was performed in 11 adult sheep at 2 levels (L2-3 and L4-5) using 3D PEEK and 3D Ti cages filled with autograft with posterior bilateral pedicle screw fixation. Fusions were evaluated at 8 and 16 weeks via manual palpation, microcomputed tomography (microCT), histology, and histomorphometry.

**Results:**

All animals recovered well following surgery with no adverse events. The radiolucent nature of PEEK allowed the fusions to be evaluated using radiographs and microCT. The 3D Ti cages however appeared solid rather than porous in the radiographs and presented artifacts in the microCT scans which precluded definitive determination of the fusions. Range of motion results improved with time for 3D PEEK and 3D Ti while no differences between designs were detected. Histology and histomorphometry confirmed 3D PEEK and 3D Ti supported fusion in this model using autograft.

**Conclusions:**

Range of motion and histology results were similar for 3D PEEK and 3D Ti. Radiographs and microCT could be used to assess the fusions with 3D PEEK due to the radiolucent nature. 3D Ti appeared solid in the radiographs and had image artifact in microCT which precluded definitive evaluation of the fusions. 3D PEEK and 3D Ti cages both support interbody fusion in this preclinical model.

## Introduction

Interbody fusion is an important surgical option for a variety of spinal pathologies with constant evolution in surgical approaches and techniques [[1], [2], [3]], imaging modalities [[4], [5], [6], [7]], graft materials [[8], [9], [10]], implant designs and materials [[11], [12], [13], [14], [15], [16]].

Additive manufacturing, a vast topic in itself and by no means a new technique, offers unique opportunities in implant design that are either too difficult or cost-prohibitive with traditional manufacturing methods or even impossible with metals [[17], [18], [19], [20]] or polyetheretherketone (PEEK) [[21], [22], [23], [24], [25], [26], [27], [28], [29]]. Clearly, there will be many more options in the future for clinicians to consider with respect to the choice of material, design, and manufacturing methods.

PEEK was introduced as a material for interbody fusion devices during the 1990s [30]. The elastic modulus of PEEK is more closely matched to cortical bone compared to Titanium alloys (Ti) reducing stress shielding [31]. PEEK is radiolucent which allows radiograph evaluation overcoming the disadvantages of solid titanium and its alloys, whilst delivering equivalent rates of fusion [4,32] Additive manufacturing has enabled the mass commercialization of titanium interbody devices with complex geometries for bone ingrowth [[33], [34[, [35]] as well as reducing stiffness and stress shielding compared with solid titanium designs [36].

PEEK’s relative inertness has often been associated with limited osseointegration in interbody fusion [30,37], though there is growing debate over the relative contribution of surface chemistry, nano-, micro- and macro-topography to this phenomenon [[38], [39], [40], [41], [42]]. In parallel to the emergence of additive manufactured titanium implants, there have been multiple efforts to develop porous PEEK for improved osseointegration and implant fixation. These have predominantly relied upon porogen space fillers which are subsequently leached creating porous scaffolds [[43], [44], [45], [46], [47]], but suffer to direct porous architectures in desired locations. Advancements in additive manufacturing technologies including fused filament fabrication (FFF), has driven research on porous PEEK and porous PEEK composites to improve osseointegration [23,25,[48], [49], [50]] whilst retaining the imaging benefits of PEEK. In vitro and preclinical studies have demonstrated enhanced osteogenic differentiation and fixation with surface porous PEEK technologies [51], and early clinical outcomes have been promising [52,53].

Functional large animal models, including ovine lateral interbody fusion [[33], [34[, [35],^42^,[54], [55], [56], [57], [58], [59], [60]] can be used to evaluate new materials and cage designs to better understand how new technology can improve clinical outcomes. High resolution microcomputed tomography, range of motion and histology endpoints allow detailed examinations to critically assess the biology and biomechanics of fusion.

Previous animal and clinical studies comparing osseointegration, fusion (radiographic and range of motion) and subsidence of additive manufactured titanium cages with PEEK cages have used solid PEEK cages as the reference [28,29,35,59]. The current study is the first to directly compare the in-vivo response of an additive manufactured porous PEEK (3D PEEK) and titanium alloy (3D Ti) cages. The null hypothesis was that there would be no differences in interbody fusion results based on radiographic, mechanical and histological endpoints.

## Material and methods

### Cages

**Fig. 1:**
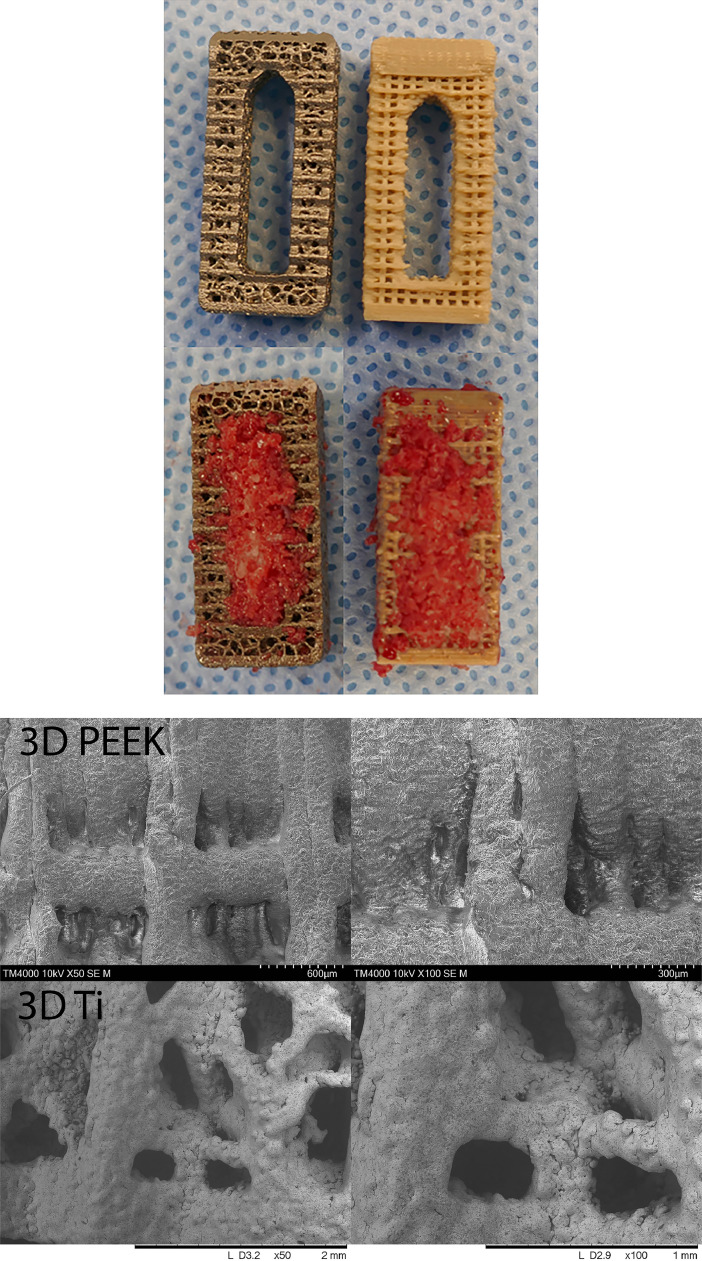
Axial views of 3D porous Ti and 3D porous PEEK cages with and without iliac crest autograft. (A) Scanning electron microscopy images for 3D printed PEEK and Ti at 50× and 100× magnification demonstrating the surface porosity and topography.

Custom sheep interbody cages (5 mm, 20 × 10 mm) designed for a lateral approach were manufactured (Fig. 1). The 3D PEEK cages were printed with PEEK-OPTIMA™ LT1 (Invibio Ltd., Thornton Cleveleys, UK) via a proprietary material extrusion technology (Bond High Performance 3D Technology B.V., Enschede, NL) where PEEK was extruded through a heated nozzle to build the part layer by layer on the print bed in a high-temperature environment. Following printing, 3D PEEK cages were deburred with a water-soluble blast media and cleaned in an ultrasonic bath with 70% isopropyl alcohol. Inferior and superior endplates consisted of 0.45 × 0.50 mm pores, lateral walls 0.44 × 0.75 mm pores and inner walls of the graft chamber incorporated pores of dimension 0.44 × 0.45 mm.

The 3D Ti cages were manufactured by SpiTrex 3D, (Carlsbad, California) using Ti-6Al-4 V ELI powder on a Renishaw RenAM 500Q additive manufacturing system. The 3D Ti cages underwent hot isostatic pressing (HIP), bead blasting, and cleaning in an ultrasonic bath and passivation. The 3D Ti cages incorporated a Voronoi lattice structure (beam thickness 0.30 mm) with ∼58% open porosity. All cages gamma sterilized at 25 kGy (Steritech, Wetherwill Park, NSW). Scanning electron microscopy images are provided in Fig. 1A demonstrating the surface topography of the 3D PEEK and 3D Ti cages.

### Study design

A nonconsecutive 2 level model (L23 and L45) was performed in eleven 4–5-year-old ewes with 6 animals allocated to 8 weeks and 5 animals allocated to 16 weeks.

### Surgical model

Eleven Border Leicester Merino Cross (4–5 years old) were enrolled following institutional ethical clearance (Approval # 21/114A) using an endplate sparing model [33,34,42,54,60] .

Transdermal fentanyl patches 24 hours before surgery for pre-emptive analgesia [61] and to provide smoother sedation and anesthetic induction. Animals were sedated with an intramuscular (IM) injection of Xylazine (0.2 mg/kg) followed by Ketamine IM (6 mg/kg) 15 minutes later. One gram of Cephalothin (18−22 mg/kg mg/kg) was given intravenously and 5 mL oxytetracycline (200 mg/mL) at 10–12.5 mg/kg IM. Benacillin (Procaine penicillin 150 mg/mL) 1 mL/10 kg was given IM. The transdermal fentanyl patches were replaced to provide a minimum of 72 hours of postoperative analgesia and Carprofen (Rimadyl 50 mg/mL) at 3–4 mg/kg IM given before surgery. Anaesthesia was maintained using isoflurane (1.5%–3%) and oxygen (2 L/ min) throughout the procedures.

Cancellous bone autograft was harvested from the left iliac wing and finely morselised with a ronguers and packed into the cage apertures. The lateral interbody surgical procedure began with blunt finger dissection and psoas muscle retraction anteriorly to expose the annulus. The annulus was transected with a #15 scalpel blade. The lateral annulus and nucleus pulposus were removed 2- and 3-mm pituitary rongeurs. The endplates were carefully prepared using a mini-Cobb elevator and a rasp to remove remaining soft tissue and maintain endplate integrity. The annulus was released at the far side using a #15 scalpel. Two 4.5 mm pins were placed in the cranial and caudal vertebral bodies and the level distracted. The cages were implanted using a custom inserter, which protected the cages and prevented autograft loss during impaction. The cages were inserted, and depth confirmed with fluoroscopy and the retractors released. The soft tissues and the skin were closed in layers with resorbable sutures.

The animals were repositioned in the prone position and re-draped for pedicle screw fixation. A dorsal mid-line skin incision over the L23 and L45 levels was made. The paraspinal muscles were elevated and lateralised with a mini-Cobb elevator to expose the facets and transverse processes, allowing insertion of pedicle screws (5 × 40 mm) and rods (5.5 mm) bilaterally at L23 and L45. The soft tissues and the skin were closed in layers with resorbable sutures.

Radiographs were taken postoperatively to confirm cage and screw positions in the posteroanterior plane. Animals were group housed in the acute postoperative period (2 weeks) in a climate controlled indoor facility and outdoor paddocks thereafter. The general demeanor, health and incision sites were monitored throughout the in-life phase.

### Radiographic, range of motion, and histological endpoints

Animals were euthanized at 8 and 16 weeks after surgery. The fusion assessments included gross dissection, Faxitron X-rays, manual palpation, microCT, nondestructive robotic range of motion, PMMA histology, and histomorphometry [33,34,42,60].

The lumbar spines from L1-L6 were harvested and inspected for any adverse reactions. The spines were radiographed in the posteroanterior and lateral planes with pedicle screws and rods remaining. The rods were removed and the stability of the L23 and L45 motion segments assessed by manual palpation in lateral bending and flexion-extension by 2 blinded trained observers. The mobility of the untreated level (L34) was used as a relative comparison. The fusions were graded as either fused (rigid, no movement) or not fused (not rigid, movement detected). Manual palpation grading results were examined with a Kruskal-Wallis nonparametric test using IBM SPSS Statistics (version 27) with significance set at p < .05.

The spines were radiographed again in the posteroanterior and lateral planes with the rods removed. The L23 and L45 motion segments were isolated and microCT performed using an Inveon Scanner (Siemens, USA) (54 microns). The micro-CT reconstructions were evaluated by 2 blinded observers reviewing the coronal and sagittal planes to assess fusion status and any adverse reactions.

The motion segments were prepared for nondestructive robotic range of motion [33,42,60]. Four 4 × 15 mm screws were inserted into the vertebral bodies to assist in potting in a resin for testing. Range of motion in flexion—extension (FE), lateral bending (LB) and axial rotation (AR) was performed using a Denso Robot (Denso, Kariya, Aichi, Japan) and SimVitro software (Cleveland Clinic, Cleveland, Ohio, USA). A 7.5 Nm pure moment was applied in FE, LB and AR and angular deformation recorded. Each profile was repeated 3 times and a mean value obtained for each level. Data was analyzed using a 2-way Analysis of Variance and a Games Howell post-hoc test with significance set at p < .05 when appropriate using IBM SPSS Statistics (version 27).

The posterior elements were removed with a band saw and higher resolution microCT scans performed (38 microns) prior to phosphate buffered formalin fixation and PMMA histology [34,42,60,[62], [63], [64]]. Serial sagittal sections (∼15 microns, minimum of 3 per sample) were taken with a Leica SP1600 Microtome (Leica Biosystems, Nussloch, Germany). The sections were briefly etched in acidic ethanol (98 mL ethanol 96% and 2 mL HCl 37%) and stained with methylene blue followed by basic fuchsin. The staining results in bone staining pink, fibrous tissue blue/purple. Histology was reviewed blinded to time, as the cage type was apparent, to examine endplates status, tissue integration into the porous domains of the cages and aperture. The cage-bone interface and local reactions were examined at higher magnification for the presence of inflammatory cells.

Histomorphometry was performed on low images (1.25×, 1 mm scale bar) using a validated custom MATLAB program (Version 3.3) to determine the area percentage bone tissue (new bone and marrow), and other soft tissues present. This analysis included the walls of the cage and aperture. The regions of interest (ROI) were defined using a polygon technique. The graft bone tissue (mineralized bone and bone marrow elements) was identified by pixel color and morphology and the area reported as a percentage of the ROI. Mean values were obtained for each animal. Analysis of variance (ANOVA) and a Games Howell post hoc test were performed with significance set at p < .05 using IBM SPSS Statistics (version 27).

## Results

All animals recovered well following surgery with no adverse events throughout the in vivo phase. All wounds were well healed, and the gross harvest did not reveal any adverse reactions.

**Fig. 2:**
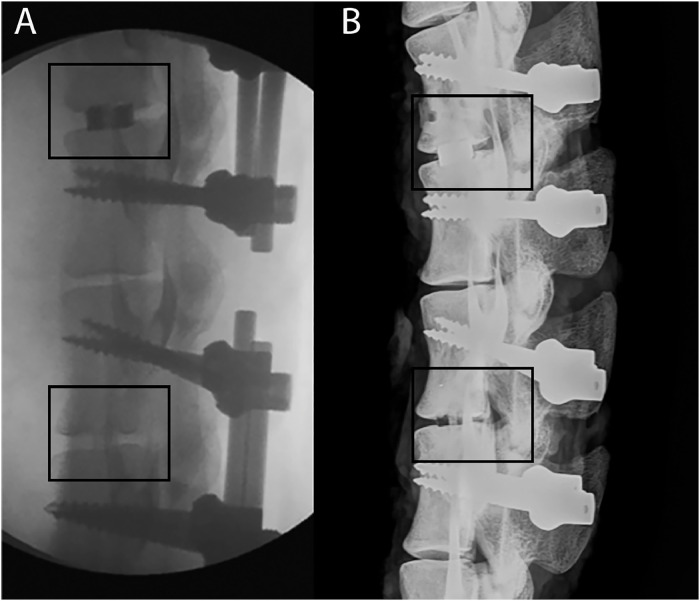
Lateral intra-operative fluoroscopy (A) and Faxitron radiograph (after the rods were removed) at 8 weeks (B). The top level (L23) was implanted with a 3DTi cage filled with autograft which can be visualised due to the radio-opacity of titanium. The bottom level (L45) was implanted with a 3DPEEK caged filled with autograft. The 3DPEEK cage cannot be detected while the graft at the time of surgery is noted as well as a bony bridge at 8 weeks.

**Fig. 3:**
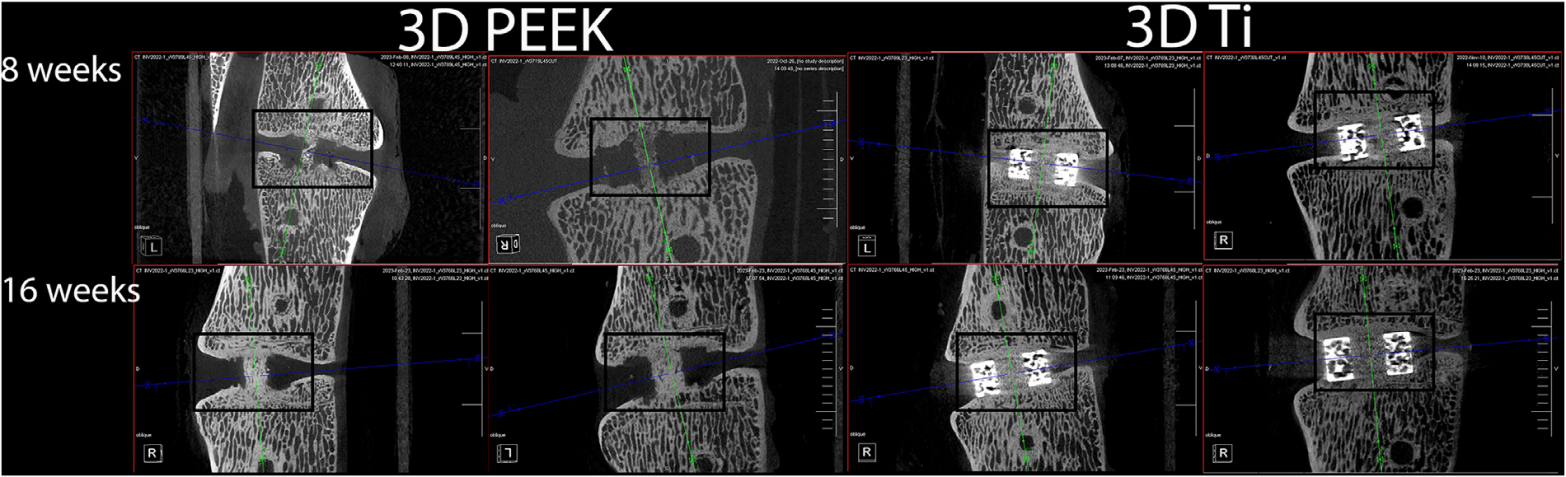
Examples of sagittal micro-computed tomography images (38 microns) for 3DPEEK and 3DTi at 8 and 16 weeks. The 3DPEEK cages are not visible while the progression in bone fusion within the aperture as well as osseointegration into the cage can be seen in the microcomputed tomography scans. The 3DTi cages were noted along with image artifact that precluded any definitive determination of bone within the aperture as well as within the porous regions of the 3DTi cage.

The 3D PEEK cages were not visible in the radiographs and allowed assessment of the fusion without any imaging artifact (Fig. 2). Integration into the porous domains and host vertebral bodies with the 3D PEEK cages was discernible using microCT (Fig. 3). On the other hand, radiographic assessment of aperture fusion or vertebral body integration was of little value for the 3D Ti cages (Fig. 2). The 3D Ti cages appeared solid rather than porous in the radiographs. Visualization of any bone in the aperture or any potential host bone integration with 3D Ti was not possible using high-resolution Faxitron radiographs. The 3D Ti presented artifacts in microCT which precluded definitive determination of the bone (Fig. 3). Endplate resorption was noted with a 3D Ti cage at 8 weeks in radiographs and microCT. No other adverse reactions were noted.

No differences were detected between 3D PEEK or 3D Ti cages with time in lateral bending, axial rotation or flexion-extension at 7.5 Nm (Table 1, p > .05). Time was a significant factor for 3D PEEK and 3D Ti cages with statistically significant reductions in lateral bending (p = .008) and flexion-extension (p = .006) for 3D PEEK and 3D Ti in lateral bending (p = .017) and flexion-extension (p = .031). No differences were detected in axial rotation with time (p > .05).

**Table 1:** Range of motion data (mean ± standard deviation)

Group	Weeks	Sample size	Manual palpation	Lateral bending	Axial rotation	Flexion-extension
3D PEEK	8	6	3 of 6	7.55 ± 1.46*	3.86 ± 0.95	6.59 ± 1.30^^^
3D PEEK	16	6	5 of 5	4.09 ± 0.62*	3.34 ± 0.89	3.22 ± 0.71^^^
3D Ti	8	5	4 of 5	6.11 ± 1.19^⁎⁎^	3.49 ± 1.65	6.88 ± 1.98^^^^
3D Ti	16	5	5 of 5	3.68 ± 0.87^⁎⁎^	3.36 ± 1.56	3.15 ± 0.97^^^^

No differences were detected in manual palpation results between 3D PEEK and 3D Ti at 8 or 16 weeks.

Time was a significant factor for 3D PEEK and 3D Ti with statistically significant reductions in lateral bending and flexion-extension for 3D PEEK (*p = .008, ^^^p = .006) as well as for 3D Ti in lateral bending and flexion-extension (^⁎⁎^p = .017, ^^^^p = .031).

**Fig. 4:**
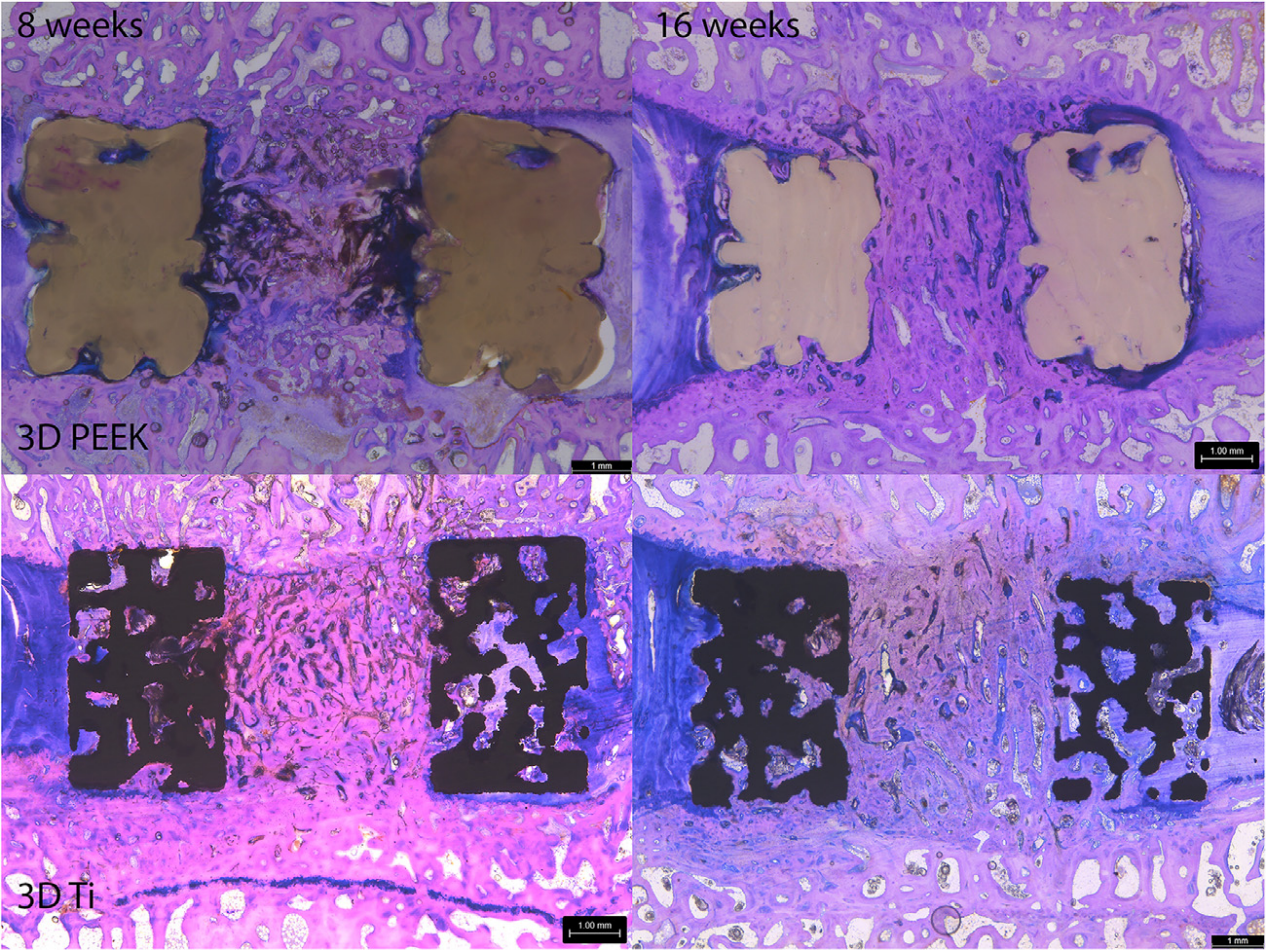
Sagittal plane histology stained with methylene blue and basic fuchsin for 3DPEEK and 3DTi at 8 and 16 weeks. A progression in graft maturity within the aperture was noted with 3DPEEK and 3DTi demonstrating both cages supported fusion. The 3DTi cages were more porous compared to the 3DPEEK. Both cages supported areas of osseointegration in the porous domains of the cage.

Histologically, 3D PEEK, and 3D Ti cages performed in the same manner supporting new bone formation in the aperture and integration with the host vertebral bodies that improved with time (Fig. 4). Autograft incorporated to the host end plates coupled with remodeling with time with both cages. The 3D Ti remained intact with no evidence of damage whereas the PEEK cages presented some deformation that was presumed to have occurred at the time of implantation. Time was an important variable in healing of the newly formed tissues within the aperture porous cage walls. The tissue within the aperture was a combination of residual graft material, newly formed bone, fibrocartilage and some fibrous tissue. The tissue within the porous walls of the 3D Ti cage was fibrous and, in some instances, new bone.

Bone within the aperture increased between 8- and 16-weeks with a reduction in other tissue as the fusions progressed for both cages but was only significantly different for 3D PEEK (Table 2, p = .035) while no differences were detected with 3D Ti cages between 8- and 16-weeks (p = .151). No difference was detected for bone in the aperture between 3D PEEK and 3D Ti cages at 8 weeks (p = .385) or 16 weeks (p = .227). The 3D Ti supported more bone within the porous regions of the wall compared to 3D PEEK at 8 weeks (^p = .008) and 16 weeks (^^p = .001) which reflects differences in the amount of material present between the designs. Bone in the available void (BIAV) increased with time for 3D Ti (**p = .037) and was statistically greater than 3D PEEK at 16 weeks (p = .003).

**Table 2:** Histomorphometry data (mean ± standard deviation)

Group	Weeks	Region of interest	Bone	Other tissue	Implant	Bone in available void
3D PEEK	8	Aperture	0.47 ± 0.12*	0.52 ± 0.11		
3D PEEK	16	Aperture	0.64 ± 0.11*	0.35 ± 0.10		
3D Ti	8	Aperture	0.56 ± 0.21	0.44 ± 0.21		
3D Ti	16	Aperture	0.71 ± 0.05	0.29 ± 0.05		
3D PEEK	8	Cage	0.02 ± 0.01^^^	0.09 ± 0.02	0.89 ± 0.02	0.17 ± 0.12
3D PEEK	16	Cage	0.02 ± 0.01^^^^	0.10 ± −.01	0.88 ± 0.02	0.13 ± 0.06^#^
3D Ti	8	Cage	0.08 ± 0.05^^^	0.38 ± 0.05	0.54 ± 0.02	0.18 ± 0.10^⁎⁎^
3D Ti	16	Cage	0.14 ± 0.03^^^^	0.31 ± 0.04	0.55 ± 0.02	0.31 ± 0.07^**,#^

Bone within the aperture increased with time and was significantly different for 3D PEEK (*p = .035). The 3D Ti design supported more bone formation within the porous regions of the cage compared to 3D PEEK at 8 weeks (^^^p = .008) and 16 weeks (^^^^p = .001). Bone in the available void increased with time for 3D Ti between 8 and 16 weeks (^⁎⁎^p = .037) and was statistically greater than 3D PEEK at 16 weeks (^#^p = .003).

## Discussion

There remains great interest in improving clinical outcomes for all surgical procedures. Additive manufacturing, while not a new technology, offers advantages over traditional manufacturing in creating complex shapes. Additive manufacturing using titanium alloys in orthopaedics has reported for many years [65] with interest in improving osseointegration through design [17,18,66] and more recently with PEEK [11,[23], [24], [25],^67^,^68^].

This study evaluated interbody fusion using 3D printed spinal cages made from PEEK or Ti alloy. An established endplate sparing interbody fusion model in adult sheep was used [54] combined with posterior fixation using bilateral pedicle screws [33,34,42,60] with a variety of endpoints. The surgical technique and appropriately sized cages for the anatomic space allowed for a more clinically relevant model for comparing these interbody fusion technologies.

Our findings demonstrate additively manufactured porous PEEK and Ti cages support interbody fusion. Time was an important factor with the fusions improving between 8 and 16 weeks in both groups. The 3D PEEK and 3D Ti performed in the same manner from a surgical, postoperative recovery, manual palpation, range of motion and histological points of view.

The Faxitron radiographs and microCT demonstrated differences between 3D PEEK and 3D Ti. These results reaffirmed the imaging artifact, and limitations present with Ti compared to PEEK to monitor bone formation and fusion [[4], [5], [6], [7],^69^] regardless of it being porous or not. The 3D Ti cages were visible in all radiography modalities compared to 3D PEEK. While the 3D Ti cages were porous, this was not apparent in plain films or Faxitron radiographs where they appeared as solid blocks. MicroCT demonstrated the 3D Ti was porous, while a definitive assessment of the bone in the aperture or the walls was not possible due to artifact. In contrast, the radiolucent nature of 3D PEEK allowed for determination of fusion. These results are not surprising considering the known shortcomings with Ti and metal artifact [70,71].

The 3D Ti cages exhibited significant metal artifact on CT imaging, extending beyond simple high signal intensity. This artefact manifested as complex variations in signal patterns and texture within the porous structure, potentially mimicking trabecular bone or presenting as a diffuse grey haze. Notably, these artefacts were not confined to the porous regions but also affected the aperture. Consequently, relying solely on radiographic assessment may lead to misinterpretation, suggesting bone ingrowth where histological analysis confirms the presence of soft tissue. This potential for misinterpretation of bone due to metal artefact could contribute to observed differences in fusion rates between PEEK and Ti cages [72,73] particularly in studies relying solely on radiography.

Histology (Fig. 4) and histomorphometry (Table 2) demonstrated 3D PEEK and 3D Ti cages supported fusion. While the cages had similar gross dimensions, there are differences in material (PEEK vs Titanium) and porosity within the walls. Iliac crest cancellous autograft supported new bone in the aperture that improved with time without any intervening fibrous tissue for both designs. New bone was also found in the porous walls of the devices and in some cases not in contact with the host vertebral body. This may preclude this bone from contributing to mechanical stability and load transfer. The walls of the 3D Ti were more porous than the 3D PEEK. This porosity in the 3D Ti cages supported more bone formation compared to 3D PEEK cages (Table 2). Bone in the available void, which accounts for differences in the porosity, increased with time for 3D Ti and was statistically greater than 3D PEEK at 16 weeks (Table 2).

The lumbar sheep spine is well accepted for biomechanical and preclinical studies as a model of humans [[74], [75], [76]]. The sheep lateral interbody fusion model is often used to evaluate interbody cages and bone graft technologies. This includes models where the endplates are removed, due to size of the interbody cages, and bilateral posterior pedicle screws [55,59], or fixed with a single rod laterally [35,57] or where anatomically more suitable cages are used with supplementary fixation using a single rod laterally [54] or with bilateral posterior pedicle screws [33,34,42,60] as used in this study. Collectively, these studies examine a wide range of topics aimed at understanding fusion including solid PEEK versus 3D Ti alloy cages [35,59], porous Ti composite cages [57], the effect of implantation site [60], dose effects of BMP-2 [54] and role of macrotopography features on a titanium coated PEEK cages [42]. The current study extends this conversation to include a direct comparison of 3D PEEK to 3D Ti.

This preclinical study is limited considering a single design of 3D Ti and 3D PEEK 3D cages were examined along with short follow-up and a single graft material (autograft). Considering the wide range of design options with additive manufacturing, these results need to be interpreted with caution. The geometric design and metal volume can influence image artefact and the ability to confidently report radiographic progress with standard clinical imaging. The mechanical properties of 3D PEEK cages must also be taken into consideration as some deformation of the PEEK cages was observed upon histological review. This was presumed to have occurred at the time of implantation but was not detectable radiographically. Finally, the microCT used to assess bone and fusion represents a best-case scenario under controlled laboratory conditions. Yet under these circumstances the authors were still unable to definitively determine aperture fusion or bone in the 3D Ti despite the porous nature.

The potential clinical significance of the current study calls into question the accuracy of fusion assessment with radiographs or CT scans when using Ti compared to PEEK regardless of manufacturing method. Differences in radiographic signals and potential artefacts can influence accurate or objectively monitoring. The radiolucent nature of PEEK allows for a definitive radiographic assessment. Additive manufacturing implants with PEEK or Ti offers a wide range of design options that requires careful consideration prior to clinical use.

## Funding

This study was funded by InVibio Ltd.

## Declarations of competing interests

William R Walsh, PhD is an Editorial Board Member for the Journal of Orthopaedic Research, Clinical Orthopaedics and Related Research, Biomaterials, American Journal of Sports Medicine and was not involved in the editorial review or the decision to publish the article. Matthew Pelletier, PhD is an Editorial Board Member for the Journal of Spine Surgery and was not involved in the editorial review or the decision to publish the article.

The authors declare the following financial interests/personal relationships which may be considered as potential competing interests:

William R Walsh, PhD: Institutional Funding received from Invibio and consulting.

Royalties from SeaSpine, Seaspine consulting, Scientific advisory board Kuros.

William R Walsh, PhD is an Advisory Reviewer for the Spine Journal as well as on the Editorial Board member: Journal of Orthopaedic Research, Clinical Orthopaedics and Related Research, Biomaterials, American Journal of Sports Medicine.

Matthew Pelletier, PhD: Royalties from SeaSpine.

Matthew Pelletier is an Associate Editor of the Journal of Spine Surgery.

Dan Wills, PhD BScVet– No known competing financial interests or personal relationships.

Tian Wang, PhD- No known competing financial interests or personal relationships.

Max Lloyd, BScVet **-** No known competing financial interests or personal relationships.

Michael Veldman, BS – is an employee of Invibio.
